# Challenges in early phase of implementing the 1-3-7 surveillance and response approach in malaria elimination setting: A field study from Myanmar

**DOI:** 10.1186/s40249-020-0632-7

**Published:** 2020-02-10

**Authors:** Poe Poe Aung, Zaw Win Thein, Zar Ni Min Hein, Kyaw Thet Aung, Nwe Oo Mon, Nay Yi Yi Linn, Aung Thi, Khin Thet Wai, Thae Maung Maung

**Affiliations:** 1 Duke Global Health Institute Myanmar Program, Yangon, Myanmar; 2 Department of Medical Research, Ministry of Health and Sports, Yangon, Myanmar; 3 National Malaria Control Program, Department of Public Health, Ministry of Health and Sports, Nay Pyi Taw, Myanmar

**Keywords:** Malaria elimination, 1-3-7 approach, Surveillance and response, Basic health staff, Mixed methods, Myanmar

## Abstract

**Background:**

The National Plan for Malaria Elimination (NPME) in Myanmar (2016–2030) aims to eliminate indigenous *Plasmodium falciparum* malaria in six states/regions of low endemicity by 2020 and countrywide by 2030. To achieve this goal, in 2016 the National Malaria Control Program (NMCP) implemented the “1-3-7” surveillance and response strategy. This study aims to identify the barriers to successful implementation of the NPME which emerged during the early phase of the “1-3-7” approach deployment.

**Methods:**

A mixed-methods study was conducted with basic health staff (BHS) and Vector Born Disease Control Program (VBDC) staff between 2017 and 2018 in six townships of six states/regions targeted for sub-national elimination by 2020. A self-administered questionnaire, designed to assess the knowledge required to implement the “1-3-7” approach, was completed by 544 respondents. Bivariate analysis was performed for quantitative findings and thematic analysis was conducted for qualitative findings using Atals.ti software.

**Results:**

Although 83% of participants reported performing the key activities in the “1-3-7” surveillance and response approach, less than half could report performing those activities within 3 days and 7 days (40 and 43%, respectively). Low proportion of BHS correctly identified six categories of malaria cases and three types of foci (22 and 26%, respectively). In contrast, nearly 80% of respondents correctly named three types of case detection methods. Most cited challenges included ‘low community knowledge on health’ (43%), ‘inadequate supplies’ (22%), and ‘transportation difficulty’ (21%). Qualitative data identified poor knowledge of key surveillance activities, delays in reporting, and differences in reporting systems as the primary challenges. The dominant perceived barrier to success was inability to control the influx of migrant workers into target jurisdictions especially in hard-to-reach areas. Interviews with township medical officers and the NMCP team leaders further highlighted the necessity of refresher training for every step in the “1-3-7” surveillance and response approach.

**Conclusions:**

The performance of the “1-3-7” surveillance and response approach in Myanmar delivers promising results. However, numerous challenges are likely to slow down malaria elimination progress in accordance with the NPME. Multi-stakeholder engagement and health system readiness is critical for malaria elimination at the sub-national level.

## Background

The World Health Organization (WHO) has announced an ambitious goal of global malaria elimination by 2030. Specifically, it aims to reduce malaria cases by 90% compared to those reported for 2015 [1]. The success of this agenda largely depends on the readiness of health systems within individual countries to support global efforts. Effective use of diagnostic microscopy, point-of-care rapid diagnostic test (RDT), and easy access to potent antimalarial drugs by the frontline health workers have enabled most of the progress achieved towards this goal to date [1, 2]. Although Africa continues to present the primary battle ground for malaria worldwide, roughly 1.6 billion people in Asia and the Pacific Region were reported to be at risk of malaria infection in 2017 [3]. Myanmar, one of the countries in the Greater Mekong sub-region, carries a disproportional malaria burden for Asia both in terms of malaria prevalence as well as morbidity and mortality due to the disease [3]. However, Myanmar has shown a remarkable progress in reducing malaria morbidity and mortality over the past decade (79% reduction in cases and 99% reduction in deaths in 2016 compared to 2005) and is on track for malaria elimination by 2030 [4–6].

The strategy for achieving malaria elimination in Myanmar, detailed in the National Plan for Malaria Elimination (NPME) in Myanmar (2016–2030), focuses the interruption of transmission and elimination of indigenous *Plasmodium falciparum* (*Pf*) malaria in six states/regions of low endemicity by 2020 with a further commitment to countrywide malaria elimination by 2030 [6]. The success of this strategy, however, hinges on a strong country-wide surveillance and response system identified as one of the pillars of The Global Technical Strategy for Malaria Framework (2016–2030) [7–9]. The so called “1-3-7” approach describes a system of activities dependent on timely diagnosis and response to malaria cases including: 1) case notification within 1 day, 2) case investigation and classification within 3 days, and 3) foci investigation and response within 7 days of RDT-positive. The “1-3-7” approach has been successfully deployed by the Asia Pacific Malaria Elimination Network (APMEN) and was first implemented in China [9–11]. In 2016, Myanmar National Malaria Control Program (NMCP) adopted this strategy as “Alert-Audit-Act” approach to enable robust surveillance in line with the strategy outlined in the operational manual for malaria surveillance in elimination settings [7–9, 12, 13].

Assessment of health system readiness for global malaria response in elimination settings also requires a deeper insight into human resources training and capacity [14, 15]. In the context of primary health care in Myanmar, basic health staff (BHS) includes midwives, public health supervisors-2, and village health volunteers. Midwives are generally responsible for maternal and child health and immunizations in rural settings and frequently deal with malaria diagnosis and treatment in pregnant women and childhood illness. Public health supervisors-2 (PHS-2) are responsible for other disease control measures in rural settings unrelated to child and maternal care. Village health volunteers serve as the first point of contact in all issues pertaining to village-level health care and disease reporting. In contrast, Vector Borne Disease Control Program (VBDC) staff work at the township level and are in charge of control measures for prevention and elimination of vector borne diseases, including malaria. BHS and VBDC staff are the key personnel at the frontline in implementing the “1-3-7” approach to surveillance while continuously challenged by resource scarcity and limited access to remote rural populations [7, 15, 16].

Several previous studies, which were undertaken in China – the first country to operationally deploy the “1-3-7” approach, have reported a range of implementation challenges, including timeliness of case reporting, case classification, insufficient support at the village level, challenges in vector control measures, transportation difficulties in hard-to-reach areas, access to mobile migrant populations, and complexity of establishing multisectoral collaboration, faced by malaria experts, epidemiologists and health workers [9–11]. A recent quantitative study from Myanmar conducted in 2016 reported early findings on challenges faced by Myanmar health care professionals; however, as an “early results study” it was able to analyse the results only in three out of six low endemic states and regions of Myanmar targeted for malaria elimination by 2020 [8]. In this present study, we aim to broaden the scope to include results from all six states and regions currently targeted for malaria elimination as we believe that this information is critical for addressing the existing programmatic gaps in supporting the effective health system infrastructure. Therefore, this study aimed to identify:

1. the level of competency in background knowledge and understanding of methods required to carry out malaria elimination and surveillance at the sub-national level;
2. perceived challenges and proposed solutions posed by frontline health workers in the early phase of implementing the “1-3-7” surveillance and response approach in malaria elimination in Myanmar.

## Methods

### Study design

This study was conducted using a mixed-methods approach [17] that integrates qualitative and quantitative methods to examine information extracted from self-administered questionnaires, Key Informant Interviews (KII), and Focus Group Discussions (FGD). The data collection was performed in six townships between October 2017 and March 2018.

### Study setting

#### General setting

The Republic of the Union of Myanmar is located in South-East Asia and borders Bangladesh, India, China, Laos, and Thailand. Myanmar is administratively divided into 14 states/regions and Nay Pyi Taw Council Territory. The geographical settings vary greatly across the country with central regions dominated by open plains which are surrounded by mountainous terrain along the perimeter including extensive remote forested sparsely populated areas with very limited access and coastal zones along southern and western borders. Myanmar’s total population in 2014 was 51.4 million with 70% of people residing in rural areas [18].

#### Specific setting

**Fig. 1:**
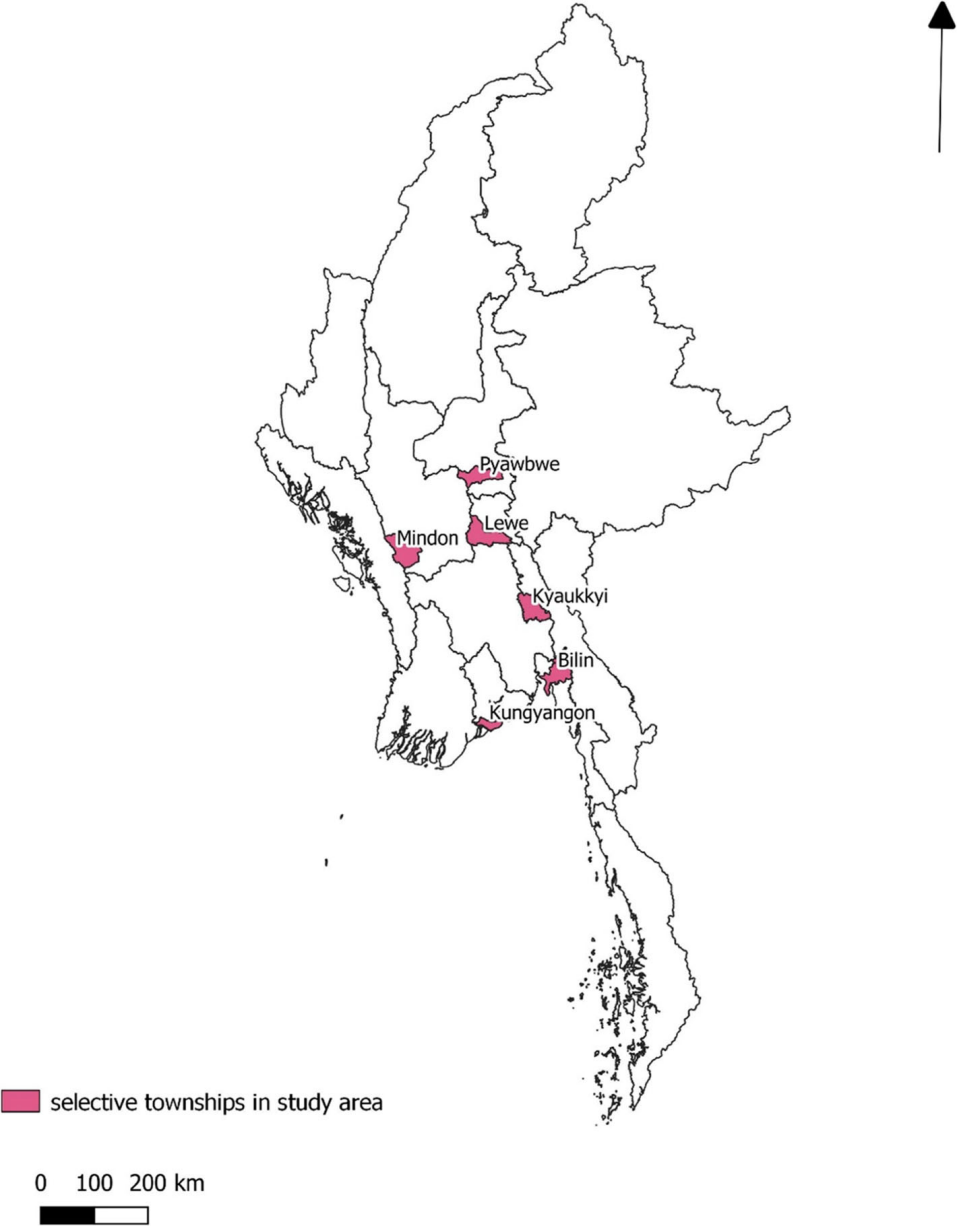
Map of six selected study sites in six states/regions Kungyangon Township in Yangon Region, Kyaukkyi Township in Bago Region, Mindon Township in Magway Region, Pyawbwe Township in Mandalay Region, Lewe Township in Nay Pyi Taw Union Territory, and Bilin Township in Mon State

**Fig. 2:**
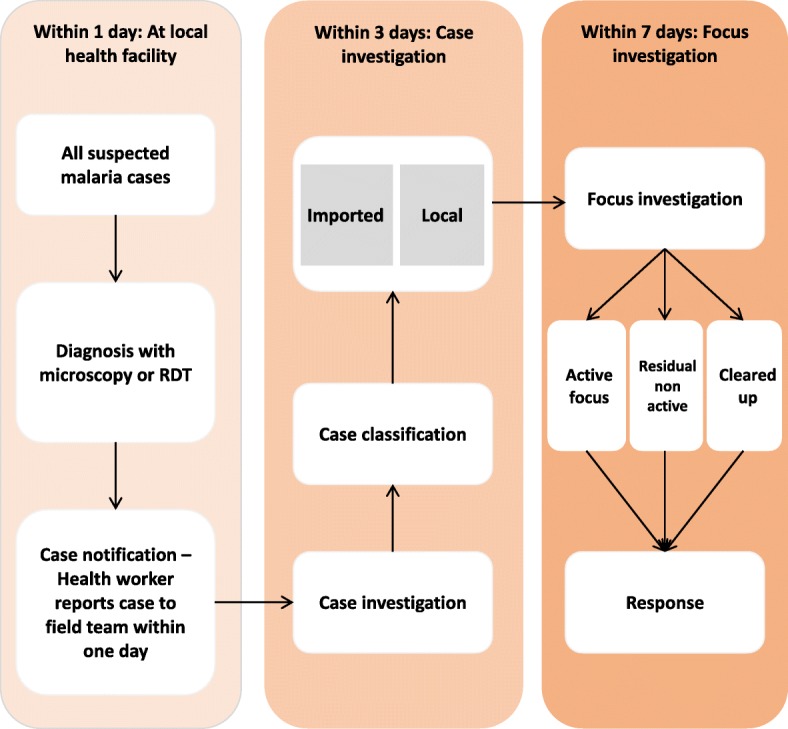
“1–3-7” approach for malaria surveillance activities implemented by National Malaria Control Program Source: Malaria Surveillance in Elimination Settings: An Operational Manual 2018 RDT: Rapid Diagnosis Test

According to the 2014 Myanmar National Census, 25.7 million people reside in 144 townships within six administrative units (states of regions) characterized by the low malaria endemic settings including Yangon, Bago, Mandalay and Magway Regions, Mon State and Nay Pyi Taw Union Territory (Fig. 1) [18]. In 2016, the annual parasite index (API) within five of these regions was less than 1 and slightly over 1 in Mon State [19]. The “1-3-7” strategy of Myanmar NMCP that comprehends malaria surveillance activities towards targeted elimination was presented in Table 1 and Fig. 2.

**Table 1:** “1-3-7” surveillance and response strategy for malaria elimination adapted by National Malaria Control Program

“1-3-7” surveillance and response strategy for Malaria Elimination in Myanmar	
1. *Case reporting within 1 day*: Any confirmed and suspected malaria cases must be reported within 24 h of diagnosis by the BHS (PHS-2 from sub-center/ midwives) and VHV.	
2. *Case Investigations within 3 days*: All malaria cases should be confirmed and visited, where the case is reported within 3 days, to determine whether the case originated (local or imported). The case investigation team consists of BHS (PHS-2 from Sub-center) and VHV (NMCP+NGO) with the support of BHS (HA/PHS-2 from RHC) and Township VBDC staff.	
3. *Focus investigation and response within 7 days*: The focus investigation should be conducted as soon as possible. If the local transmission is possible or confirmed, targeted actions to seek out other infections and reduce the chance of onward transmission is completed within 7 Days where the patient resides and/or works. The foci investigation and response team consist of the township/district/States/Regional level malaria focal persons (malaria inspector, laboratory technician, entomological staff, BHS and respective VHV.	

*BHS* Basic Health Staff, *PHS-2* Public Health Supervisor-2, *VHV* Village Health Volunteer, *NMCP* National Malaria Control Program, *NGO* Non-government Organization, *HA* Health Assistant, *RHC* Rural Health Center, *VBDC* Vector Born Disease Control Program

#### Representative types of frontline health workers involved in targeted malaria elimination in Myanmar

**Fig. 3:**
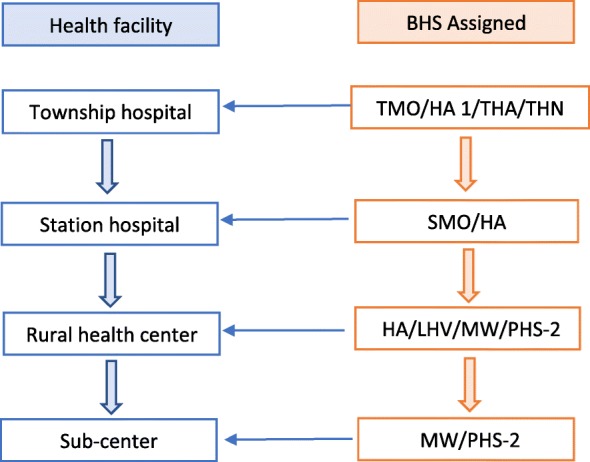
Structure of Township health system: level of health facility and basic health staff assigned BHS: Basic health staff, THN: Township health nurse, THA: Township health assistant, TMO: Township medical officer, SMO: Station medical officer, HA: Health assistant, LHV: Lady health visitor, MW: Midwife, PHS: Public health supervisor

**Fig. 4:**
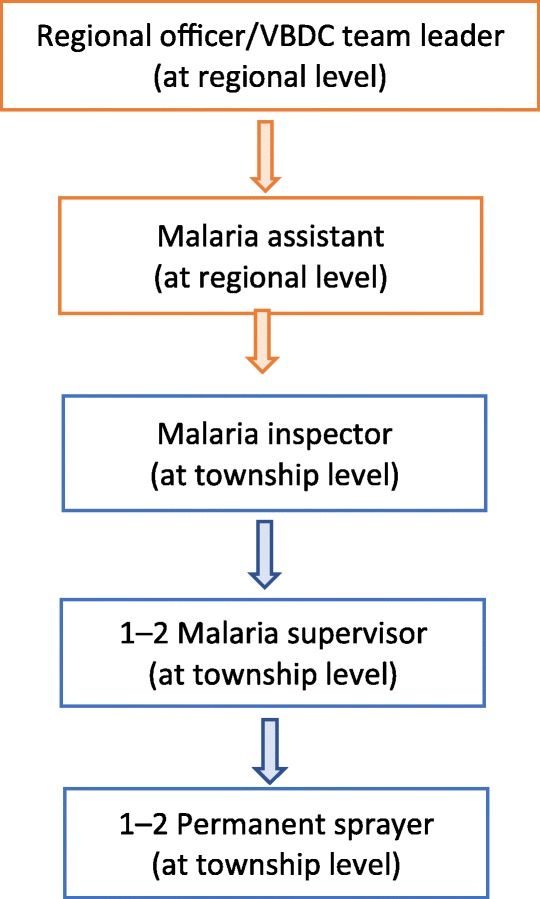
Structure of regional and township Vector-borne Diseases Control Program team

The NMCP is implementing malaria elimination activities across the country as a whole covering 330 townships within all states and administrative regions [5]. Malaria is considered endemic in 291 of the 330 townships with some of the most challenging cases for elimination found in sporadic hotspots of malaria occurrence within very low endemicity areas. Reflecting the variety of geographical and administrative settings found across Myanmar, there is a range of frontline healthcare workers who are involved in surveillance and response to malaria cases. At the village level, the BHS, including health assistants (in-charge of a Rural Health Centre), lady health visitors (who supervise the midwives and PHS-2), midwives (responsible for maternal and child health and immunization), and PHS-2 (responsible for implementing other disease control measures), are responsible for diagnosing malaria and treatment services. They also refer severe malaria cases to the nearest health facility operated by medical doctors. At the township level, VBDC staff is comprised of a malaria inspector (in-charge of township level VBDC team), a malaria supervisor (responsible for vector borne disease control measures and assisting malaria inspector) and a permanent sprayer (assisting malaria inspector and responsible for insecticide spraying). At the regional level VBDC regional officers/team leaders and malaria assistants serve as overall technical experts and supervise VBDC teams in the state/region. The structure of township level healthcare system and the assigned BHS in different level of health facilities in the rural areas and the structure of VBDC team at the township and state/regional levels are described in Figs. 3 and 4 respectively.

#### Activities of NMCP in malaria elimination

As the frontline service providers, BHS deliver primary health care to population in rural areas and direct and oversee services provided by village health volunteers. This group of healthcare workers was the primary focus of the “training the trainers” program, launched by the National Malaria Expert Group at the federal and regional levels in 2016 and 2017. The majority of the training was delivered to the township level health workers without a written manual. This study, which was conducted in the late 2017 early 2018 during the early phase of implementing the “1-3-7” approach, aimed to assess knowledge retention and service delivery preparedness of those who received the original training. The current standard operating procedures manual– Operational Manual for Malaria Surveillance in Elimination Settings with an emphasis on the “1-3-7” approach– was introduced later in 2018 and thus its impact is not reflected in the outcomes of this study [5, 6, 13].

### Study population

The quantitative component included information collected using self-administered questionnaires from all eligible BHS and VBDC staff in the study townships. The qualitative component focused on analysing information provided by team leaders/regional officers from NMCP, VBDC staff, township medical officer (TMO), and other BHS during KIIs and FGDs.

### Sampling and sample size

We randomly selected six out of 70 townships from six states/regions (one township per state/region) where the capacity building “train the trainers” program was implemented between 2016 and 2017. These included Kungyangon Township in Yangon Region, Kyaukkyi Township in Bago Region, Mindon Township in Magway Region, Pyawbwe Township in Mandalay Region, Lewe Township in Nay Pyi Taw Union Territory, and Bilin Township in Mon State.

#### Quantitative component

A total of 544 BHS and NMCP/VBDC staff who were available at the time of survey from six selected townships were recruited following an informed consent.

#### Qualitative component

A dimensional sampling method was applied to cover both township and regional level healthcare workers. Four out of six townships were randomly selected for the qualitative component. Three VBDC team leaders/regional officers, one VBDC staff and six TMOs were recruited for KIIs. FGDs included 7–9 discussants per session and were conducted in each of the four selected townships to include the total of 42 discussants. The interviews were conducted at a private location to ensure the confidentiality of the information collected.

### Data collection, variables and data sources

#### Quantitative component

Trained surveyors introduced a pre-tested self-administered structured questionnaire to all available respondents (*n* = 544) at various health facilities. The original questionnaire included 20 knowledge and opinion items. Key variables in the questionnaire analysed for this study covered 17 of those items which focused on three primary topics: 1) key surveillance activities for malaria elimination, 2) knowledge of methods for case detection and type of foci investigation, and 3) participants’ opinion on the likelihood for achieving targeted malaria elimination in their areas. Operational definitions of key variables used are provided in Table 2**.**

**Table 2:** Operational definition of case detection and key surveillance activities

Key surveillance activities	Operational definition
Case detection	
Passive case detection	Detection of malaria cases among people who go to a health facility or a community health worker (CHW) on their own initiative to get treatment, usually for fever
Active case detection	Detection by health workers of malaria cases in the community and in households, sometimes among population groups who are considered to be at high risk
Reactive case detection (RACD)	RACD is triggered by the identification and notification of an index case. After the investigation and classification of the index case, RACD may be implemented within the household of the index case, or over a radius around the household or within the whole focus.
Malaria cases	
Indigenous case	A case contracted locally with no evidence of importation and no direct link to transmission from an imported case
Introduced case	A case contracted locally, with strong epidemiological evidence linking it directly to a known imported case (first-generation local transmission)
Imported case	Malaria case or infection in which the infection was acquired outside the area in which it is diagnosed
Relapse case	Malaria case attributed to activation of hypnozoites of *Plasmodium vivax* or *P. ovale* acquired previously.
Induced case	A case the origin of which can be traced to a blood transfusion or other form of parenteral inoculation of the parasite but not to transmission by a natural mosquito-borne inoculation
Recrudescent case	Recurrence of asexual parasitemia of the same genotype(s) that caused the original illness, due to incomplete clearance of asexual parasites after antimalarial treatment
Type of foci	
Active foci	A focus with ongoing transmission
Residual non-active foci	Transmission interrupted recently (1–3 years previously)
Cleared foci	A focus with no local transmission for more than 3 years and which is no longer considered residual non-active

Source: “malaria-surveillance-monitoring-and-evaluation---a-reference-manual” (link: https://www.who.int › docs › default-source › documents › publications › gmp)

#### Qualitative component

Trained interviewers used a pre-tested guideline for KIIs to survey the current activities of BHS and gauge their readiness to implement the “1-3-7” surveillance and response approach to malaria elimination.

Trained moderators and note-takers arranged FGD and free-listing, as well as priority ranking exercise for selected BHS to gather the information collectively and to obtain the group consensus of their self-rated opinion. A total of 10 KIIs in six selected townships and at the state/regional level and four FGDs in four out of six selected townships were conducted. Participants were informed about the purpose of the study before data collection. Only selected participant(s) and the researchers were present during the FGD/KII sessions at a private location. The FGDs lasted on average 50 min while KIIs lasted on average 40 min. The interviews were audio-recorded as well as documented by note-takers.

### Data analysis and statistics

#### Quantitative component

Double data entry and validation were carried out within the EpiData environment (version 3.1 EpiData Association, Odense, Denmark). Data management and analysis was done using STATA software (version 14.0, College Station, Texas USA). Absolute numbers and proportions were used to describe correct answers to knowledge-based questions on malaria elimination and surveillance. The STROBE guideline for observational studies in Epidemiology was used to conduct the quantitative study and report its results [20].

The calculation of ‘Knowledge of case detection type’ required the participants to list all three types of case detection (passive, active, and reactive case detection) to be considered as “answered correctly” (Code = 1). In cases where at least one type was listed incorrectly or missing, the response received Code = 0. ‘Knowledge of malaria case categories’ required listing all six categories of malaria cases (indigenous, introduced, imported, relapse, induced and recrudescent cases). Code = 1 was assigned to responses which correctly identified all six types of malaria cases; if any of the case types were incorrectly identified or missing, the responses received Code = 0. ‘Knowledge of foci type in foci investigation’ required listing all three types of foci (active, residual non-active, cleared foci): Code = 1 was assigned to responses which correctly identified of all three types of foci, alternatively Code = 0 was assigned. ‘Knowledge of the “1-3-7” strategy surveillance activities’ included listing activities to be undertaken within 1 day, within 3 days and within 7 days of case identification. Code = 1 was assigned for correctly listing any one of the “1-3-7” strategy activities; if all three were listed incorrectly, the response received Code = 0.

#### Qualitative component

Audio recordings from FGDs, KIIs, free listing and priority ranking exercises were transcribed and typed in Myanmar language within one week after completion of data collection. Transcripts in Myanmar language were coded with the aid of ATLAS.ti software (version 6.1.1, Scientific Software Development GmbH, Germany). Two individual researchers completed the coding independently to ensure coding accuracy. Codes were discussed with the co-investigators and any discrepancies in coding by individual researchers were resolved through discussion and referring to the original audio files whenever necessary. The resultant coded transcripts underwent thematic analysis. The COREQ guidelines were used for reporting qualitative research findings [21].

## Results

### Social and demographic characteristics

#### Quantitative component

A total of 544 frontline health workers (BHS and VBDC staff) completed the self-administered questionnaire (median age 28 years [IQR: 23–38]). The majority (88%) hold a university degree. Nearly half of the respondents (46%) were midwives. Eleven VBDC staff (2%) participated at the township level.

#### Qualitative component

During qualitative interviews, 10 key informants (mean age 39.9 years, ranging between 27 and 59) participated in the discussions. They represented an equal number of male and female participants, all of whom completed university degrees and have served on average 15.3 years (range 1.5–28) as health care workers. Altogether 42 BHS (18 males and 24 females) participated in FGD (mean age of 27 years, ranging between 21 and 48), all participants attained a university degree.

### Knowledge of malaria elimination activities

**Fig. 5:**
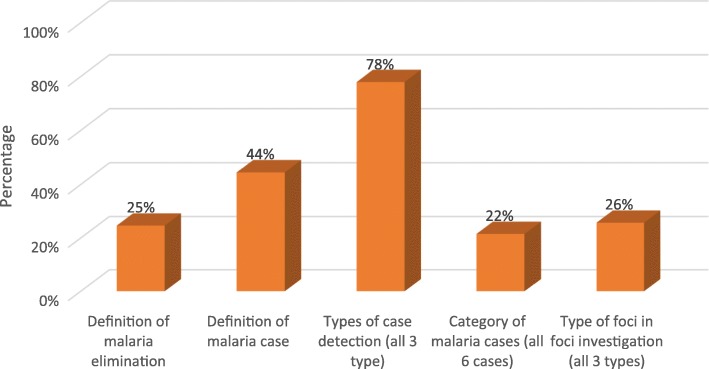
Correct Knowledge of BHSs and VBDC staffs on elimination targeted malaria surveillance activities in six selected townships for malaria elimination in Myanmar, 2017–2018

The summary of correctly answered questions covering the knowledge base for malaria elimination surveillance activities was presented in Fig. 5. There was a substantial difference in proportion of participants who correctly knew definitions of malaria case types and malaria elimination approaches (45% vs 25%). Only 22 and 26% of BHS correctly identified all six categories of malaria cases and all three types of foci, respectively. In contrast, nearly 80% of respondents could correctly state all three types of case detection methods.

### Knowledge of the “1-3-7” surveillance and response approach

Approximately 83% of participants self-reported that they knew the key activities in the “1-3-7” surveillance and response strategy **(**Table 3**)**. Their expressed knowledge about activities to be undertaken within 24 h reached the highest proportion (95%). However, less than half could detail activities to be undertaken within 3 days and within 7 days (40 and 43%, respectively) indicating limited knowledge of full surveillance strategy. These quantitative findings regarding the knowledge base related to surveillance and response in study sites are further supported by the results of the qualitative analysis presented below.

**Table 3:** Knowledge of basic health staff and Vector-borne Diseases Control Program staff on “1-3-7” surveillance and response approach of malaria elimination in six selected townships for malaria elimination in Myanmar, 2017–2018

Knowledge on activities of “1-3-7” strategy	*N*^a^	(%)
Total	453	
Within 1 day: At local health facility	434	(95)
Diagnosis with microscopy or RDT	223	(51)
Case notification	46	(11)
Treatment	4	(1)
Diagnosis and Case notification	61	(14)
Diagnosis and Treatment	65	(15)
Case notification and Treatment	2	(0.5)
Diagnosis, Case notification and Treatment	33	(8)
Within 3 days: Case investigation	183	(40)
Case investigation	178	(97)
Case classification	1	(1)
Case investigation and classification	4	(2)
Within 7 days: Focus investigation	194	(43)
Focus investigation	101	(52)
Response	61	(31)
Focus investigation and Response	32	(16)

^a^Only those who answered YES in having knowledge of activities on 1-3-7 approach

### The perceived role of BHS in the “1-3-7” approach

During a KII, one TMO described BHS as essential for field implementation of malaria elimination and as key players for response in action by closely working with village health volunteers. Interestingly, PHS-2 required more encouragement to be involved in the “1-3-7” approach, compared to midwives.

Implementing the real time case notification and case investigation for patients with positive RDT results emerged in discussions as one of the major challenges for BHS in deploying the “1-3-7” approach in the field. It was also noted that BHS play an important role in coordinating with the local community those activities that must be carried out within 7 days in line with the “1-3-7” approach.

### The role of VBDC staff in the “1-3-7” approach

Qualitative findings further revealed the key role of VBDC staff in all steps of the “1-3-7” approach. Once BHS become aware of RDT-positive cases, they are required to relay this information immediately to the regional VBDC team. Within 3 days from notification, VBDC staff visit the patient’s residence and perform case investigation/case classification by using a standard case investigation form. Within 7 days, VBDC staff assist the regional VBDC team in foci investigation and response activities. The regional VBDC team in-charged of carrying out specific response activities within 7 days, including foci investigation which covers: 1) locating a likely breeding site of female *Anopheles* mosquitos and identifying their points of access to human habitat environment, 2) administering RDT to all persons within 20 households closest to the identified positive case (the hot spot), and 3) providing health education to the community.

“*… .. we (VBDC staff) are the key players as well as the connectors. When we get the data from the village, we inform TMO and the regional VBDC team and do all the rest of the work …*” *(KII with VBDC staff).*

**Within 1 day:** KII with VBDC staff identified limited access to mobile reception as the primary barrier for timely (within 24 h) reporting of malaria positive cases by BHS and volunteers. The “1-3-7” approach was particularly challenging to implement in hard-to-reach areas with limited human resources. Subsequently, a delay in reporting RDT-positive cases by international non-governmental organizations (NGO) working at the local area resulted in delaying activities typically conducted within 3 and 7 Days.

“*… ...reporting within 24 hours is most difficult for those area where mobile reception is not good. One out of seven volunteers cannot report on time due to lack of mobile reception and compromised security for travel …*” *(KII with VBDC staff).*

A KII with VBDC staff further revealed the existence of a different reporting system for BHS and village health volunteers in some townships where pilot intervention for malaria case-based reporting (MCBR) application using mobile phone was implemented. Information entered into the MCBR app is uploaded directly to the centralized server and bypasses the township or regional level VBDC teams. Data inconsistences between carbonless paper-based report and MCBR app-based records, including potential data duplication, differences in reported details, and missing data, were also highlighted by discussion groups as a challenge in implementation of “1-3-7” approach. Mobile text message reporting, deployed by some of the international NGOs, was not implemented by NMCP.

“*… if volunteers found the RDT-positive case, he or she entered the data into tablets which directly reached the server at the central level without any input to us. Thus, we cannot enter that data into our system to avoid duplication. But we need to fill in the case investigation form. It creates confusion in the data system … …*. *a more straightforward method should be developed ……*” *(KII with VBDC staff).*

During FGDs, BHS also reported limited transportation as a major barrier for timely reporting of RDT-positive cases using paper-based forms. However, they identified electronic reporting via Facebook, Messenger or Viber as a viable solution to the transportation issue. Likewise, the idea of electronic reporting system using mobile application was preferred by a team leader during a KII.

“*… it is difficult to send the paper-based report to the regional office from the township in time within 3 days. But we can do it by Facebook or Messenger or Viber similarly to how we currently send reports for dengue fever case notification, if that is allowed …*” *(FGD with BHS).*

**Within 3 days:** BHS suggested that case investigation form was unnecessarily lengthy. Questions regarding malaria history covering the period of three previous years are not likely to yield accurate descriptions. Instead, to avoid recall bias from the patients, BHS preferred to ask malaria history limited to the span of 1 year. The complexity of the case investigation form made further decision regarding case classification prohibitively difficult even for supervisors (health assistant /lady health visitor) and VBDC staff in the fields. A team leader during a KII additionally suggested that paper-based case investigation form should be converted to a mobile-app friendly format to simplify reporting.

“*…*. *Case investigation form should be compact … .it usually takes about 30–45 min … ..when we asked malaria history from 3 years, the patient couldn’t remember it. We are wondering - is it okay to ask the malaria history from just past year?” (FGD with BHS).*

**Within 7 days:** Findings from the KII with a VBDC team leader highlighted the important role of BHS in coordinating the response activities with the local community. Local authorities in villages require continuous comprehensive updates regarding the surveillance activities to ensure strong administrative support.

“*… Administrative support plays a key role here …*. *BHS link us with the local community and local authorities in order to conduct activities of the “1-3-7” strategy on time” (KII with team leader).*

### Challenges for targeted elimination strategy by 2020

The most common barriers for malaria elimination are identified by the respondents as the ‘low community knowledge regarding health and well-being’ (43%), followed by ‘inadequate supplies’ (22%), and ‘transportation difficulty’ (21%) (Table 4**)**.

**Table 4:** Barriers and suggestions of basic health staff and Vector-borne Diseases Control Program staff on targeting malaria elimination in 2020 for six selected townships in rural area in Myanmar, 2017–2018

Barriers and suggestion	*N*	(%)
Total	544	
Barriers for malaria elimination		
Community low knowledge on health	233	(43)
Inadequate supplies	117	(22)
Transportation difficulty	115	(21)
Mobile/migrant (or) conflict affected populations	98	(18)
Financial barrier	82	(15)
Non-compliance to treatment	72	(13)
Not relevant	67	(12)
Lack of stakeholder engagement	56	(10)
Over-burden of basic health staff	51	(9)
Inadequate health education	43	(8)
Inadequate training and refresher training	24	(4)
No barrier	22	(4)
Language barrier	18	(3)
Inadequate preventive and control measures	15	(3)
Others	12	(2)
Suggestion for malaria elimination		
Enhance preventive and control measures	218	(40)
Ensure adequate support	189	(35)
Conduct training and refresher training	145	(27)
Increase health education	114	(21)
Strengthen surveillance system	83	(15)
Increase human resource	56	(10)
Improve stakeholder engagement	43	(8)
Improve effective treatment	29	(5)
Not relevant	23	(4)
Improve monitoring and supervision	7	(1)

A TMO viewed the inability to control the influx of migrant workers into their jurisdiction especially in hard-to-reach areas as the main challenge for malaria elimination. It was estimated that the majority of positive cases were emerging from plantation sites in mountainous areas and border areas.

“*… migrant population in hard-to-reach areas working for gold mining or plantation are the major challenge for me. They could not even access other health services with BHS, like immunization. Transportation is bad … .no car or motorcycle, no mobile phone network, people with very limited health knowledge, including malaria … how can I guarantee malaria elimination in 2020 for such areas …*? *?” (KII with TMO).*

### Suggestions to improve the surveillance strategy for malaria elimination

Top suggestions that emerged from this study include ‘enhancing preventive and control measures’ (40%), ‘ensuring adequate financial support’ (35%) and ‘conducting training and refresher training’ (27%). These were further supported by qualitative findings. Interviews with TMOs and team leaders elucidated that additional training and refresher training for all BHS was required at every step for the successful implementation of the “1-3-7” approach. At the same time, wider advocacy for malaria elimination with local authorities should be encouraged. In addition, coordination of activities and collaboration among different organizations or departments working on malaria is viewed as critical for effective implementation. Moreover, adequate support in terms of human and financial resources are identified as essential for malaria elimination readiness at the township level.

“*… refresher training is needed more frequently from state/region level. There is an instruction for when to do and what is needed for the township VBDC team, but it is not very effective. Better come to the field, find out the real situation and take action more effectively …*.” *(KII with TMO).*

## Discussion

This study was designed to be conducted within a time span of a few months from the 2-day training programs for malaria elimination deployed by the NMCP. The initial results have highlighted the roles and functions of the frontline health workers and the perceived challenges and barriers to implementation within the early phase of the “1-3-7” surveillance and response approach. Several findings stand out among the results particularly considering their impact on the overall success of the “1-3-7” approach implementation.

First, overall the knowledge-base of the frontline health workers reflects the current status of malaria distribution and occurrence as well as the effectiveness of training. It was evident that the majority of health workers could correctly identify the types of case detection (active, passive and reactive case detection) for malaria elimination. The apparent poor ability to define malaria elimination strategies was likely due to the lack of reported indigenous *Plasmodium falciparum* positive cases within the respective jurisdictions for three consecutive years [1, 9]. However, the inability to define malaria cases, identify case categories and types of foci also exposes the need for employing continuous education strategies, e.g. refresher training. The insufficient knowledge base within these aspects of malaria elimination approach may have a substantial impact on proper deployment of the necessary 3- and 7-days activities under the “1-3-7” approach.

Second, findings confirmed that BHS serve as the frontline service providers in Myanmar and are involved in implementation of all steps of the “1-3-7” approach. However, their main responsibility resides with testing all fever cases and notifying VBDC personnel of all RDT-positive cases within 24 h. As midwives are in general overloaded with primary health care responsibilities, the Ministry of Health and Sports has redefined the scope of responsibilities for different BHS groups in 2018 [22, 23]. Since 2012, PHS-2 were trained and attached to midwives in rural areas primarily in support of disease control activities, including malaria. Thus, at field level, the NMCP should engage more PHS-2 staff in the “1-3-7” surveillance and response activities in malaria elimination in the community.

Apart from BHS, this study also explored the involvement of VBDC staff at every step of the “1-3-7” approach. The foci investigation and response activities are found to be mainly carried out by regional and township VBDC focal persons, making the coordination support provided by BHS and volunteers to link VBDC personnel with local community and local governance a crucial piece in successful implementation of the 3- and 7-day activities.

Third, this study addressed the challenges in 1-, 3-, 7-day activities of health workers. The main challenge faced by health workers within the first 24 h (1-day) was inability to complete case notification in a timely manner which subsequently resulted in delaying the implementation of 3- and 7-day activities. The primary barriers for timely case notification include difficulty in transportation, limited mobile reception within remote hard-to-reach areas, and, in some cases, inability to access mobile/migrant populations. The use of SMS or other types of electronic reporting present a viable solution for situations where the case notification delay is caused by a limited access to transportation and remote hard-to-reach locations. Similarly, timely notification of VBDC staff of RDT-positive cases within mobile/migrant populations presents a substantial challenge, as increased malaria transmission is reported among returning migrant workers from border countries [9, 19, 24]. Studies established that migrant workers were highly mobile (with seasonal variation in their residency and mobility patterns), they also had less access to public health services and when they did, the utilization of those services was rather limited. The complex nature of migrant workers’ movements across Myanmar increases their vulnerability to malaria. An outreach program conducted through malaria volunteers could provide the necessary linkages to the mobile/migrant workers communities [19, 24]. Additionally, establishing check-in points and administering RDT at the border crossing areas were suggested by the study participants as a potential solution to addressing malaria transmission through migrant populations. The migrant mapping activity was also suggested as one of the essential activities to strengthen malaria surveillance and gain further understanding of the mobility patterns of migrant workers in the area. The lack of security hampered implementation of the “1-3-7″ approach in conflict-affected areas and was also identified as one of the major barriers in malaria elimination similarly to previous studies along China-Myanmar border [12, 14].

An additional challenge for the “1-3-7” approach implementation is presented by the lack of or delayed reporting of RDT-positive cases from private clinics and international NGs. In China, all positive cases are reported (with almost no exceptions) within 24 h which is a reflection of a strong law enforcement program [11, 14]. In Myanmar, however, there is no legal requirement for reporting malaria cases within the 24-h window. Therefore, it was suggested that a legal pathway should be considered as a solution for universal reporting of RDT-positive cases within 24 h. More research is needed to better understand the role of general practitioners and private sector health care providers in malaria elimination and strengthening malaria surveillance.

The lengthy and complicated case investigation form presented the main challenge for BHS and VBDC staff within the scope of 3-day activities and frequently resulted in misclassification of case categories. In general, delays in 3-day activities are a consequence of delayed reporting, difficulties in transportation, and a lack of human resources. Studies in China revealed that 98% of positive cases were reported within 3 days which was achieved primarily due to timely reporting from the field and sufficient human resource availability. Similarly, logistical and transportation difficulties also have been previously found to result in delay in surveillance activities [9, 11].

Activities within the 7-day scope were primarily led by the regional and township VBDC teams and coordinated by BHS and volunteers with local community. Again, lack of human and financial resources were identified as major factors for delay in deploying 7-day activities – a finding that is in line with previously reported results [9].

Fourth, the stakeholder engagement and community involvement were essential to perform the “1-3-7” approach, especially for 7-day activities for malaria elimination as suggested in the studies from China and Laos [9, 25]. Further research is needed to improve our understanding of stakeholder engagement and community involvement in Myanmar in the context of malaria elimination.

Finally, this study highlighted the challenges arising from the use of different reporting systems (electronic and paper-based) by BHS, international NGOs, and various administrative units across the study area. Unifying and streamlining case reporting is necessary to maintain the database at the central malaria server error- and duplication-free. The introduction of the electronic Malaria Information System (eMIS) in Thailand improved point-of-care and real-time data quality and case management [26, 27]. Similarly, replacing paper-based reporting system with a mobile reporting system in South Africa substantially shortened the time period between case detection and reporting: the frontline health works readily adopted the system primarily due to the simplicity of its use [28]. To ensure timely and effective reporting, the NMCP should strengthen the surveillance database system in Myanmar by integrating with District Health Information System 2 (DHIS2) platform and upgrading the electronic surveillance database system up to township and regional level data systems. The enhanced DHIS2 should deliver a single centralized database accessible from township level to reflect all health indicators, including malaria.

Qualitative findings further suggested that the development of a case notification system could utilize the capabilities afforded by existing mobile text messaging and/or electronic reporting by mobile applications including Facebook, Messenger, or Viber in Myanmar and that this approach should be reflected in the NMEP. The potential solutions also included the electronic data entry system by tablets and direct upload to the server. In China, a wide range of mobile and cellular networks across the country is the main driver behind the widely implemented SMS text message reporting, followed by a web-based database reporting and feedback mechanism [10, 11, 14].

As the first mixed-methods study in Myanmar, this analysis uncovered not only the gaps in early phase of the “1-3-7” surveillance and response approach for malaria elimination, but also enabled us to understand the practical challenges faced by BHS and VBDC staff as well as identify potential solutions to those challenges. Our approach to data entry and verification ensured data quality which was further validated using Epidata software for the quantitative data. The use of the self-administered questionnaire in this study may have an impact on reliability of the responses and assessing the knowledge base of respondents and thus may represent a biased representation. Another limitation was that this study was conducted during the early phase of the “1-3-7” approach before the NMCP implemented standard operation procedures of malaria elimination for BHS and does not reflect the current improvements of the “1-3-7” approach implementation in Myanmar. This study should be repeated in all malaria elimination areas of Myanmar following the updated malaria elimination strategy and standard operation procedures.

## Conclusions

Myanmar has made a strong commitment to supporting the WHO’s goal of eliminating malaria by 2030. In accordance with the NMEP, Myanmar opted to deploy the “1-3-7” approach which has been proven successful in other countries within Asia. The performance of the “1-3-7” approach in Myanmar appears promising; however, frontline health workers face numerous challenges which may slow down the progress in malaria elimination. Because BHS play a crucial role at all steps of the “1-3-7” approach, their poor knowledge on key surveillance activities could prevent the malaria elimination strategy from reaching its full potential. On the other hand, improvements in reporting format and system could boost the success rate of the “1-3-7” approach and increase confidence among basic health staff and VBDC staff. Alternative strategies are needed to combat delays in case reporting within area of limited cellular coverage and limited transportation options, i.e. in remote rural areas. In order to extend the coverage for the “1-3-7” approach, multi-stakeholder engagement in collaboration with implementing partners is critical for enhancing health system readiness supported by human resource development and financial assistance in targeted townships.

## Data Availability

Data are not available in public domain because they are currently being analysed in related papers. However, data are available with the corresponding author (PPA) and may be made available upon request at the following e-mail: poepoeaung@gmail.com.

## Abbreviations

APMEN: Asia Pacific Malaria Elimination Network
BHS: Basic health staff
FGD: Focus Group Discussion
IRS: Indoor Residual Spraying
ITN: insecticide treated nets
KII: Key Informant Interview
LLINS: Long-lasting insecticidal nets
MCH: Maternal and Child Health
NMCP: National Malaria Control Program
NPME: National Plan for Malaria Elimination
RDT: Rapid Diagnostics Test
RHC: Rural Health Centre
VBDC: Vector-borne Diseases Control
WHO: World Health Organization
